# Visual reversals and biases while observing ambiguous spinning biological motion and rigid structure-from-motion

**DOI:** 10.1371/journal.pone.0343061

**Published:** 2026-02-24

**Authors:** Leo Poom, Wilma Loogna, Edvin Carstensen

**Affiliations:** Division of Perception and Cognition, Department of Psychology, Uppsala University, Uppsala, Sweden; Psychologische Hochschule Berlin, GERMANY

## Abstract

We examined perceived reversal rates and biases as observers viewed four ambiguous, motion-defined depth asymmetric point-light stimuli: a biological motion stimulus in the form of a spinning point-light walker (PLW), a rigidly spinning human figure, a spinning half-cylinder, and a wobbling slanted cylinder. The last three are rigid structure-from-motion (SFM) stimuli. We analysed angular reversal distributions to assess perceptual biases: facing-the-viewer (FTV), convexity, and depth-symmetry biases. The PLW showed the highest reversal rate and a strong FTV bias, though responses were bimodal, some observers experienced reversals every half-turn, others rarely. The rigid human figure showed a weak FTV bias. The spinning half-cylinder resulted in an initial convexity bias, but the occurrence of reversals following the initial percept instead revealed a novel “edge-in-front” bias. The wobbling cylinder showed no angular bias, and had the fewest reversals, likely due to persistent depth asymmetry throughout its motion, and/or that the wobbling prevent adaptation-recovery cycles of neural populations tuned to opposite spinning directions. Correlation analyses revealed shared mechanisms among spinning stimuli, but not with the wobbling cylinder. These findings highlight how shape and motion jointly influence perceptual reversals, refining models of bistable perception and individual variability.

## Introduction

To investigate how the visual system reconstructs 3D distal configurations from the retinal 2D projections researchers often isolate specific sources of information, such as relative motion. Wallach and O’Connell [1] demonstrated that observers can effortlessly perceive the rigid 3D structure of spinning wire-frame objects from the motions of their 2D shadows, a phenomenon known as the kinetic-depth effect. Ullman [2] expanded on this by showing that 3D structure can be effortlessly recovered from motion alone when observers viewed parallel projections of spinning cylinders where each static view appeared as a random collection of dots, a process known as structure-from-motion (SFM). Similarly, when opposite motion directions of two sets of dots are presented, motion transparency is perceived where surfaces are segregated into separate depth layers [3,4]. In a seminal study, Johansson [5,6] demonstrated biological motion perception, where biological structures and types of activity can be effortlessly perceived solely from the motions of point-lights located at the major joints of human actors.

Here, we exploited the inherent ambiguity of various point-light stimuli: biological motion in the form of a point-light walker (PLW), a rigidly spinning human shape in a walking posture, and rigid SFM of inanimate shapes, in which the near–far relationships cannot be inferred from the stimulus alone. During prolonged inspection perceptual reversals are perceived, highlighting the dynamic endogenous and interpretative nature of visual processing. Traditionally it is believed that the main processes behind perceived reversals are neural adaptation and recovery cycles or top-down driven shifts between competing interpretations of sensory information influenced by various experiential biases and stimulus configurations [7–14], although the timing of reversals may be determined by neutral noise [15]. Biases occur when one interpretation of ambiguous stimuli is favoured, typically due to stimulus configuration and prior experiences.

**Fig 1:**
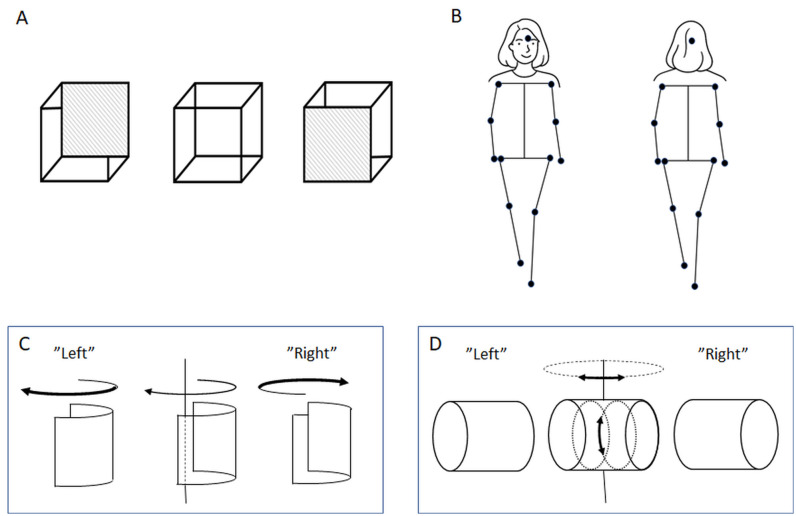
Depth ambiguities and biases. **(A)** The Necker cube in the middle with the two interpretations, where the view from above is typically preferred. **(B)** A point light walker, here illustrated by an ambiguous stick-figure, can be perceived as facing or facing away from the viewer, but a facing-the-viewer bias is typically preferred. **(C)** A SFM stimulus displaying a spinning half-cylinder is depth asymmetric in frontal views (convex or concave), but depth symmetric in side views and should then reverse more easily (possibly to uphold a convex percept). **(D)** An obliquely viewed wobbling SFM-cylinder (oscillating around the vertical and main axis) is observed with either its left or right side facing the viewer. In movie sequences of C and D only dots randomly spread on the surface of the objects are visible.

Among other inanimate motion-defined depth ambiguities beside those used here are the Lissajous figures, generated by intersecting perpendicular sinusoids animated with an increasing phase shift. These figures evoke the illusion of three-dimensional rotation in depth, resembling spinning wireframe objects, and tend to perceptually reverse when the stimulus lines overlap [7,14] at which point they are depth-symmetric. Also, spinning asymmetric SFM stimuli tend to perceptually reverse at depth-symmetric viewpoints, where the near and far parts of the object switch place without occupying new empty regions in perceived space [16]. In addition, numerous static ambiguous stimuli are well documented [17], including wireframe figures such as the Necker cube. Interestingly, neurotypical individuals tend to perceive wireframe figures with a view-from-above bias (Fig 1A), a perceptual tendency that is diminished in individuals with autism spectrum disorder (ASD), who also demonstrate lower reversal rates, suggesting a predominance of bottom-up over top-down processing mechanisms [18]. Likewise, deficits in perceiving coherent and biological motion [19] and reduced reversals in structure-from-motion stimuli [20] have been observed in individuals with ASD, linking perception of these stimuli with clinical psychology.

When viewing a biological motion stimulus in the form of a point-light walker (PLW) depicted as walking on a treadmill, roughly 80% of observers perceive the figure as facing toward them rather than away, a facing-the-viewer (FTV) bias [21] (Fig 1B). A treadmill-displayed PLW almost never undergoes perceptual reversals. In contrast, a spinning PLW can be perceptually decomposed into its spinning and walking components [22], and it reverses far more frequently than a rigidly spinning inanimate structure-from-motion (SFM) object [10,23]. This elevated reversal rate may arise from the non-uniform dot density that defines the human form. Moreover, the alternating depth-symmetric and depth-asymmetric views that occur during rotation may promote reversals at specific phases of the spinning cycle [23]. Depth-symmetric and depth-asymmetric phases are especially pronounced in a spinning half-cylinder defined by SFM, whose direction of rotation is perceptually ambiguous (Fig 1C). By contrast, an SFM cylinder that is slanted in depth and wobbles around both its main axis and its vertical axis remains depth-asymmetric throughout its motion (Fig 1D).

A previous study compared perceptual reversals between a spinning point-light walker (PLW) and a rigidly spinning human figure, both exhibiting similar depth-symmetrical properties in upright and inverted orientations [10]. The PLW elicited more frequent reversals in both conditions, but only the upright PLW, readily recognizable as biological motion, produced a facing-the-viewer (FTV) bias. This suggests that limb motion contributes to the high reversal rate in both upright and inverted PLWs, while the FTV bias is specifically triggered by the familiar configuration of the upright figure. This FTV bias may stem from a convexity bias where observers tend to direct attention on the lower part of an upright PLW [24] and interpret the knees as bulging outward, consistent with an FTV percept [25]. More broadly, ambiguous depth indentations are generally perceived as convex rather than concave, likely because most objects in the environment exhibit outward-bulging shapes.

**Fig 2:**
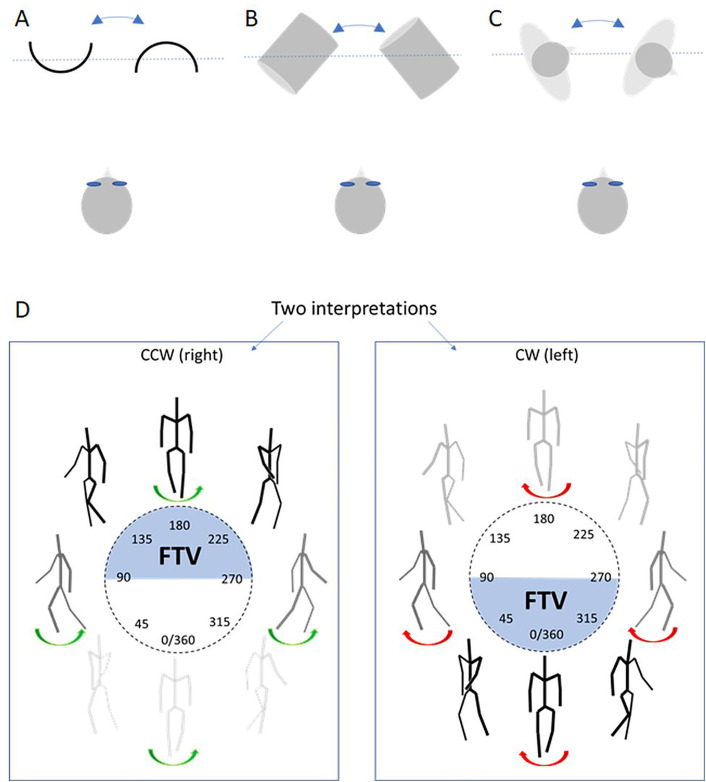
Schematic illustrations of the ambiguities. The two interpretations of (A) the spinning half-cylinder bulging toward or away from the viewer, (B) the cylinder slanted in depth, with its left or right side facing the viewer, and (C) the human figure obliquely facing away or toward the viewer. Figures (A-C) are displayed in a view from above with the frontal projection plane indicated by the dotted lines. **(D)** Schematic illustration of a stick-figure shown at 45° steps on a circular plot. The figure may be perceived as spinning clockwise (CW) or counter clockwise (CCW), with reversals in direction accompanied by depth-order reversals. Similar convex–concave depth reversals occur with the spinning half-cylinder (not shown). Perceived leftward spinning at 45° angle indicate that the figure is facing the viewer (FTV, blue shaded area within the circles, and black stick figures) whereas a perceived rightward spinning at the same angle indicate that the figure is perceived facing away (light grey stick-figures). Facing direction is not specified at side views (90° and 270°).

The convexity bias influences perceived SFM [26], and also result in the hollow potato illusion and the hollow-face illusion [27,28]. When viewing a spinning hollow face mask, it appears to reverse its spinning direction at every half turn to preserve its perceived convex shape [29]. Similar to a spinning face mask, a spinning half-cylinder (Fig 1C) alternate between bulging toward (being convex) or away (being concave) from the observer as it spins. It is likely that the convexity bias influences the perceived reversals of the spinning half-cylinder to counteract concave interpretations by flipping spinning direction as the shape enters a concave phase (Fig 2A). The FTV and convexity bias can be targeted by the timing of reversals of spinning figures. As they spin these figures alternate between facing toward and away from the viewer, and likewise between convex and concave shapes. If the FTV-bias (or convexity) bias dominates, reversals should occur at similar orientations for the human figures and the spinning half-cylinder, and if a common convexity bias is involved for the human figures and the half-cylinder, then the reversal rates should correlate.

Additionally, reversals could be facilitated at depth-symmetric perspectives for spinning figures (a depth symmetry bias) [16] which also can be targeted by the timing of reversals since the figures alternate between depth-symmetric and asymmetric perspectives. The spinning half-cylinder reverses smoothly in side views when it is depth symmetric, allowing seamless reversals. When it bulges toward or away from the observer then reversals result in rough transitions in depth where new areas in perceived depth will be occupied by the perceived object. If seamless reversals are preferred then a depth-symmetry bias will be observed. The wobbling SFM cylinder, slanted in depth, is depth-asymmetric throughout its whole motion sequence (Fig 2B), the perceived reversals are few, and not correlated with reversals obtained with other ambiguous stimuli [8,10]. The low reversal rate for the wobbling cylinder could also be due to impeded adaptation-recovery cycles of populations of units tuned to conjunctions of depth and direction. Such units are maximally adapted by symmetric uniformly spinning shapes which result in sustained activation of such units [13,30]. The human figure is maximally depth-asymmetric when viewed from oblique angles (Fig 2C), and the depth-symmetry bias therefore predicts more frequent reversals when the figure is seen from frontal, back, or pure side views.

The aims of this study are to characterize the orientation-specificity of perceptual reversals across all stimuli, to determine how facing-the-viewer, convexity, and depth-symmetry biases shape the orientations at which reversals occur for the spinning stimuli, and to assess correlations in reversal rates among the four stimuli, with particular emphasis on whether the wobbling cylinder shows any relationship to the others given its previously reported lack of association. To examine how biases affects reversal dynamics, and to examine potential shared mechanisms, we measured reversal rates and their correlations, and compared the angular distributions of reversals for the different motion defined stimuli. Observers were asked to report reversals while viewing depth-asymmetric shapes consisting of a spinning walking PLW (S1 Movie), a spinning rigid human shape in a walking pose (S2 Movie), a spinning half-cylinder (S3 Movie), and a wobbling cylinder (S4 Movie) (in the experiment a fixation cross was shown above all stimuli, but in the movies it is shown only in S3 and S4). An FTV-bias is expected for the PLW that could result from a convexity bias at the knees. The timing of reversals for the spinning half-cylinder is likely influenced by the convexity bias (as for a spinning hollow mask). The type of bias for the different stimuli should be revealed by the timing of reversals and their correlations. A previous study showed that reversal rates for an obliquely viewed wobbling structure-from-motion (SFM) cylinder (Fig 1D) do not correlate with the reversal rates of a wobbling or static wire-frame Necker cube (which are also depth-asymmetric) or a spinning SFM cylinder [8,10]. We investigated if reversal rates for the wobbling cylinder would also be unrelated to the reversal rates of the spinning stimuli used here.

## Methods

### Participants

The results from thirty-five observers (10 female), mean age 27 years (std 9.9), were used in the analyses. An additional observer was excluded from the analyses due to failure to follow instructions. They were all compensated with a cinema ticket for their participation. The experiment took 25–30 minutes.

### Ethical considerations

Data was anonymized and no sensitive personal data was collected and participants gave written informed consent. The study involved no physical intervention or biological material taken from the participants, they were neither physically nor psychologically manipulated, and faced no risk of being harmed physically or psychologically. The participants were informed that they could terminate participation at any time during the experiment and still keep the compensation. According to the Swedish act concerning the Ethical Review of Research involving humans (2003:460), ethics approval is required only if any of the above considerations are not fulfilled. Since these considerations were fulfilled, no ethics approval was required for this study. The study was conducted in accordance with the code of ethics of the World Medical Association (Declaration of Helsinki).

### Stimuli

The PLW was generated by an algorithm that simulated human walking by hierarchically coupled pendulums in 3D-space displayed on a projection screen from an arbitrary viewing perspective. The algorithm is described and available online [31]. The PLW and the rigid human shape was simulated to spin in clockwise direction, but their perceived direction was ambiguous. Fig 2D shows an ambiguously spinning stick-figure (PLW) in 45° increments. When perceived as spinning counter clockwise (CCW), the figure faces the viewer between 90°–270° and faces away between 270°–90°; the opposite holds for clockwise (CW) perception. Reversals invert both spinning direction and depth order. The orientation of the figure at which reversals occur specify whether the figure reverse to face toward or away from the observer. The range of orientations where the PLW faces the viewer when the perceived spinning direction is CCW and CW are marked by the shaded regions within the circles in Fig 2D. Equivalently, the range of orientations where the spinning half-cylinder bulges toward the viewer are marked by the shaded regions. The spinning PLW and spinning rigid human shape in a walking posture) were composed of 13 dots displayed on the main joints. The height of the figure as it appeared on the screen was 13.5 cm, and its width in a frontal view was 4.5 cm. To approximate parallel projection, a viewing distance of 7000 body lengths was employed, measured from the human point-light figure to the projection point. The projection surface was defined as a frontal plane intersecting the figure’s midsection (as illustrated by the dotted lines in Fig 2 A-C).

The vertically oriented spinning half-cylinder, bisected along its main axis, was composed of 100 dots randomly displayed on its surface, its height on the screen was 9 cm and the diameter 6 cm. It was simulated to spin in a clockwise direction but the perceived direction was ambiguous. When its direction reversed its front-back relationship also reversed (Fig 1C).

The wobbling cylinder was horizontally oriented and its main axis was oriented with an average slant of 50° in depth displayed with 200 dots randomly spread on its surface (keeping the dot density same as for the spinning half-cylinder), its height on the screen was 6.5 cm and its width 9 cm. The cylinder performed 10° amplitude oscillations around its main axis and 10° amplitude oscillations around a vertical axis at the midpoint of the cylinder. The sign of slant was perceptually ambiguous with either its left or right side facing the viewer (Fig 1D).

The fixed temporal parameters are spinning and wobbling speed, walking speed, and stimulus duration. Each stimulus completed 24 turns which took 168 sec. One turn took 7 sec, comprising 150 still in the motion sequence during which the walker completed 5 periods in the gait cycle (1.4 sec. gait period). The wobbling cylinder motion was created by sequential presentation of 50 stills completing one cycle in the motion sequence, 72 cycles were completed during the stimulus duration.

The human shape was maximally depth-asymmetric when viewed at oblique angles at 45°, 135°, 225°, and 315°, whereas in side-views and frontal-views at least the torso dots were depth-symmetric and the left and right side of the shape simply switched places upon reversals. The spinning half-cylinder was maximally depth symmetric when viewed from a side view at 90° and 270° orientation in its spinning cycle, but maximally depth-asymmetric at 0° and 180°. The wobbling cylinder was depth-asymmetric throughout its motion sequence due to its slant in depth.

Dots were slightly yellow with RGB code (250, 250, 120) presented against a black (0, 0, 0) background. A green (0, 256, 0) fixation cross was presented above each stimulus. One cm on the screen corresponded to about 1° visual angle from a viewing distance of 57 cm, which was the approximate viewing distance used. In the simulations, the figures started from 0° (facing the viewer) with simulated clockwise spinning (as seen from above, so the nearest part of the figure moved to the left).

Movie sequences of the stimuli can be viewed and downloaded in the supplementary files. The spinning PLW: S1 Movie, the spinning rigid human shape in a walking posture: S2 Movie, the spinning half-cylinder: S3 Movie, and the wobbling cylinder: S4 Movie.

### Procedure

For the spinning stimuli, the perceived near side could be perceived as moving leftwards or rightwards, when the perceived spinning direction reversed also the depth relation reversed. Similarly, reversals from the wobbling cylinder consisted in the left or right side pointing toward, or facing the viewer. Reversals were reported as “Left” when spinning direction (facing direction) reversed from right to left by pressing the ‘f’ key, and “Right” when the opposite reversal was observed by pressing the ‘k’ key. The spacebar was pressed to indicate mixed responses or when it was difficult to perceive a clear spinning direction. Observers were also instructed to focus on the fixation cross to minimize possible influences on reversals by fixation on various parts of the stimuli. It was found in pre-experimental tests that participants were sometimes confused over which percept they had experienced just before an eye-blink, especially when reversals were rare. To support memory, participants last response was indicated by a green arrow pointing left or right, or a line without any arrowhead (for a mixed response) which appeared above the fixation cross after each response. The order of stimulus presentations was randomized for each participant. Before a new stimulus, a brief instruction text appeared describing the next stimulus and the task. The study was part of an undergraduate thesis and data collection started 23/01/2024 and was finished 10/04/2024.

### Statistical analyses

Traditional p-values and if possible, Bayes factors (BFs) were calculated by a freely available software [32]. The BF provides evidence both for and against the null-hypothesis, where BF_10_ is the ratio between the likelihoods of the results given H_1_ and H_0_ (BF_01_ is the inverse ratio). As general rules of thumbs BF_10_ (BF_01_) between 1 and 3 (1–1/3) is anecdotal evidence, between 3 and 10 (1/3–1/10) is moderate evidence, between 10 and 30 (1/10–1/30) is strong evidence, between 30–100 (1/30–1/100) is very strong, and beyond that extremely strong evidence [33,34]. The default settings in JASP for the effect size priors were used. In Bayesian correlation analysis, a flat prior (also known as a uniform prior) means that all correlations (from −1 to +1) are considered equally likely for the alternative hypothesis before observing the data.

Frequentist hypothesis testing was performed for the circular response distributions (Bayes factors could not be calculated for circular data in the JASP-software). The Rayleigh uniformity test of circular data detects departures from uniformity in circular data. The Watson-Wheeler test for circular data was used to test if two samples of “responses are differently distributed around the circle. The Harrison-Kanji perform ANOVAs on circular data based on the χ^2^ statistics were applied to detect differences in mean response vectors in circular data, where the null hypothesis is an equal mean direction across groups.

For the correlation analyses the non-parametric rank correlation coefficient Kendall Tau-b (which has a range between −1 and +1) is recommended for skewed data when samples are small, or when the data contain ties and was used for the correlation analyses.

## Results

**Fig 3:**
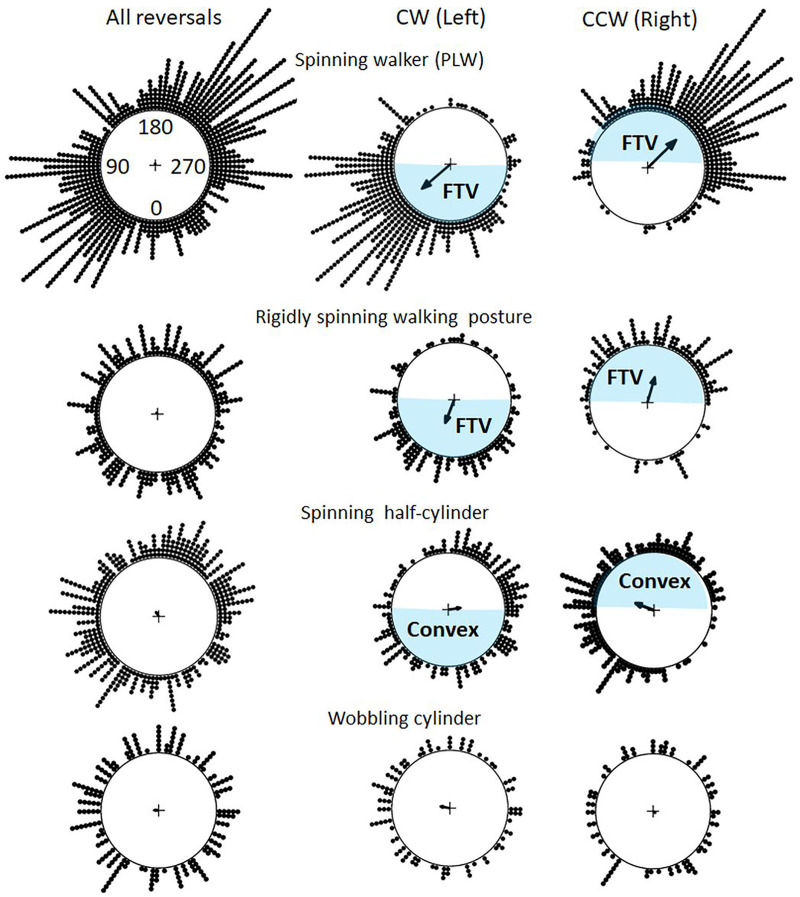
Distribution of button presses indicating reversals. Circular plots of the distributions of the reported reversals as a function of spinning angle (0-360°) during 168 sec across all participants where each response is indicated by a dot. From top to bottom: a spinning point-light walker, a rigid human shape in a walking posture, a spinning half-cylinder, and a wobbling cylinder. On the leftmost plots are distributions of all reversals. The responses are sorted as clockwise responses in the middle column (CW or left) and counter clockwise responses in the rightmost column (CCW or right). The CW responses indicate reversals from spinning rightwards to leftwards, and the CCW responses indicate reversals from spinning leftwards to rightwards. The blue shaded ranges indicate facing the viewer (FTV) responses, or bulging outward (convexity) responses for the half-cylinder, and the unmarked range indicate facing away, or bulging inward, responses. The mean direction where responses were made is displayed by the arrows, and resultant lengths are normalized to the unit circle.

The leftmost plots in Fig 3 show the angular distribution of all button presses to report reversals from all observers across all 24 turns of the spinning figures. Visual inspection reveals a clear bimodal distribution for the reversals of the PLW. The percentage unclear/mixed responses were few and not included in the following analyses (3.1%, 4%, 2.9% and 7.9% for the spinning PLW, rigidly spinning human shape, spinning half-cylinder, and wobbling cylinder respectively). The “Left” responses (near part moving leftward, or clockwise) and “Right” responses (near part moving rightward, or counter clockwise) are separately displayed in the middle and rightmost plots in Fig 3. Responses within the shaded ranges in the circular distributions are reversals indicating facing the viewer (convex), and responses within the non-shaded range are reversals indicating facing away from the viewer (concave). The distributions of the “Left” and “Right” responses departed from uniformity for the PLW and the rigid human shape as well as for the spinning half-cylinder as evidenced by the Rayleigh test (all p’s < .001), whereas the wobbling cylinder did not (p = .091 and.862 for “Left” and “Right” responses respectively). No correction of response times was applied; analyses were conducted on unadjusted raw data.

The mean angular direction where the “Left” and “Right” responses were made are displayed by the arrows in Fig 3, indicating that they were made at opposite angular directions for all spinning figures. The distributions of the “Left” and “Right” differed for both the PLW and rigid human shape as well as for the spinning half-cylinder (W = 579, W = 96.4, and 53.7, all p’s < .001, as calculated by the Watson-Wheeler test for circular data), whereas for the wobbling cylinder no difference was found (W = 3.6, p = .17).

The Harrison-Kanji ANOVA on circular data, based on the χ^2^ statistics, was applied to detect interactions (2 x 2) between the mean directions of “Left” and “Right” responses and each stimulus pairs (the PLW and the rigid human shape, the PLW and the spinning half-cylinder, the rigid human shape and the spinning half-cylinder). Few reversals were reported for the wobbling cylinder, and since they were uniformly distributed this stimulus was not included in these analyses. Interactions were found between all stimulus pairs investigated, meaning that the mean directions of the “Left” and “Right” response vectors were influenced by stimulus condition (56 < χ^2^ < 474, all p’s < 0.001). To conclude, the “Left” and “Right” reversals were made at opposite angular directions of each spinning figure, and these directions differed between the different figures.

**Fig 4:**
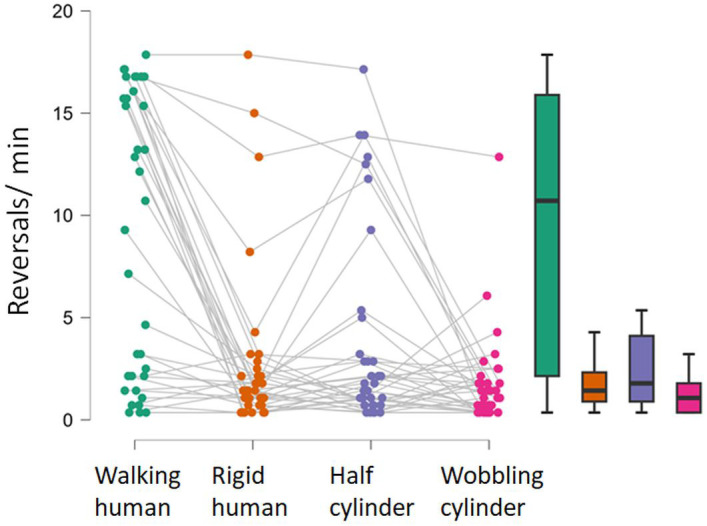
Reversal rates. Raincloud plots of reversals per minute in the four stimulus conditions, and on the right are the corresponding box-whisker plots showing the medium, the interquartile extent, and ranges excluding data beyond 1.5 times the interquartile ranges from the upper and lower bound of the boxes.

Fig 4 shows raincloud plots of the number and the corresponding box-whisker plots of reversals/minute. Inspection of the raincloud plot for the PLW indicate that the distribution of reversals is bi-modal where most observers either perceive just a few reversals per minute or over 15 reversals per minute. Reversal rates were statistically different between stimuli (repeated measures ANOVA: F(3)= 25, p < .001; BF_10_ = 4 ·10^9^). Post hoc comparisons show that the PLW (walking human) results in higher reversal rates than the other stimuli (499 < posterior odds < 14700), whereas no difference was obtained between the rigid SFM-stimuli (spinning rigid human, spinning half-cylinder, and wobbling cylinder, 0.23 < posterior odds < 1.47). An additional Friedman test was conducted due to non-normality of the data which confirmed a statistically significant difference in reversals across conditions (χ²(3) = 29.00, *p* < .001). The non-parametric Conover’s post-hoc comparison test also confirmed that the PLW results in higher reversal rates than the other stimuli (all p’s < .001, corrected by Bonferroni-Holm method), whereas all other comparisons were non-significant (.13 < p < .44).

**Fig 5:**
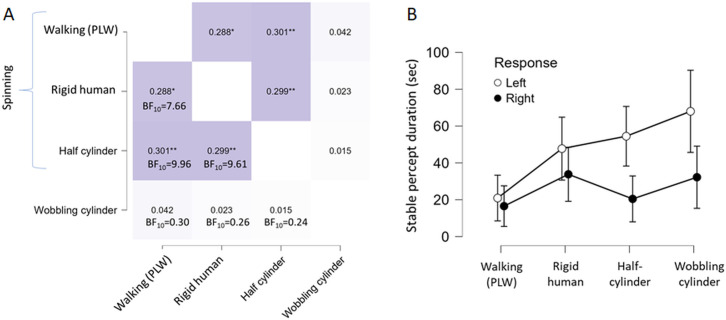
Reversal rate heatmap and stable durations. **(A)** The heatmap show the Kendall Tau-b correlations, and Bayes factors, between reversal rates for the different stimuli (* p < .05, ** p < .01, one-tailed for positive correlations). **(B)** Shown are stable durations of perceived left vs. right spinning directions. For the wobbling cylinder left vs. right refer to the cylinder perceived as facing the viewer with its left or right side.

Fig 5A is a heatmap of the non-parametric Kendall Tau-B correlations between the reversal rates in the four stimulus conditions. The Bayes-factors suggest evidence for positive correlations between reversal rates of all pairs of stimuli except the reversal rates for the wobbling cylinder which is unrelated to the other stimuli, replicating previous reports [8,10]. This indicate that a common factor is involved for all spinning stimuli. Also, the FTV scores, calculated as the differences between facing toward FTV+ and facing away responses FTV-, correlated between the spinning PLW and rigid human shape, Kendall tau-b = .27 (p = .015, BF_10_ = 5.3). No correlations between FTV scores for the spinning half-cylinder and the rigid human shape and the PLW was obtained, Kendall tau-b = .13 and.060 respectively (p = .15, BF_10_ = .66 and p = .031, BF_10_ = .34).

The mean stable percept durations are shown in Fig 5B. A two-tailed Wilcoxon tests revealed a very strong support for a left duration bias for the spinning half-cylinder (W = 509, BF_10_ = 80.2), and moderate evidence for the wobbling cylinder (W = 455, BF_10_ = 3.96). Moderate evidence for no difference (support for H_0_) was found for the rigid human shape and PLW (W = 379 and 372, BF_10_ = .276 and.273 respectively). The left duration bias for the wobbling SFM cylinder was replicated [8,10] but in the previous study no such bias was obtained for a vertically oriented spinning SFM-cylinder, whereas here it emerged for the likewise spinning half-cylinder. A common bias regarding stable left durations is thus evident for all but the PLW. At present it is not clear what causes this bias for some asymmetric rigid shapes. Also, in the previous study the left duration bias decayed in an additional set presented 15 minutes after a first set, suggesting a transient character.

**Fig 6:**
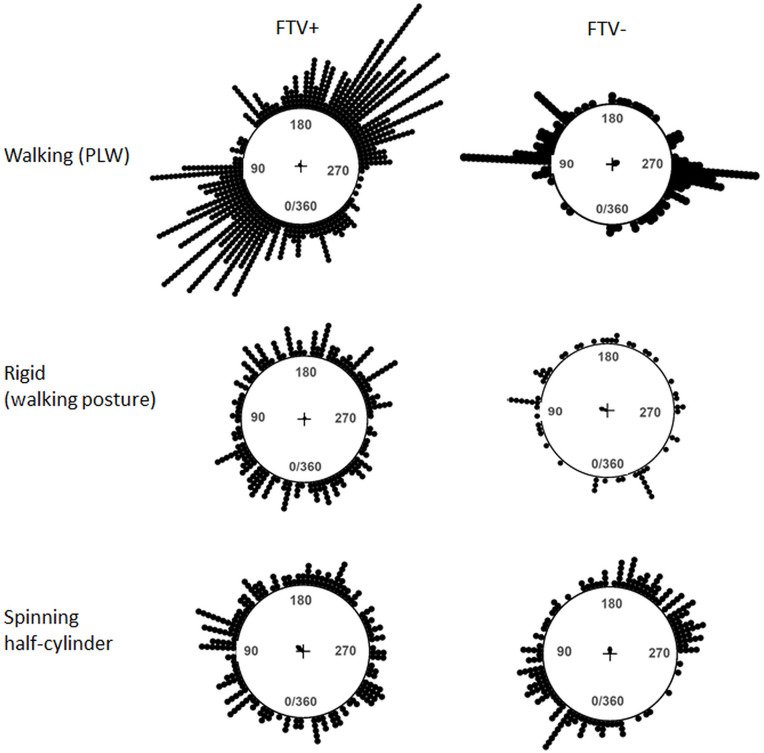
Distribution of button presses sorted as facing the viewer and facing away responses. Responses are sorted as facing the viewer responses in the left column (FTV+) and facing away responses in the right column (FTV-), across all participants where each response is indicated by a dot. For the spinning half-cylinder, FTV+ and FTV- corresponds to bulging toward (convex) and bulging away (concave) from the viewer.

Fig 6 show the angular distribution of reversals sorted as reversing from facing-away to facing-toward (FTV+) and from facing-toward to facing-away (FTV-). In other words, the CW and CCW responses within the blue shaded ranges in Fig 3, indicating reversals to facing the viewer (FTV+), are combined in the angular distributions in the left column. Likewise, the CW and CCW responses within the unmarked ranges in Fig 3, indicating reversals to facing away (FTV-), are combined in the angular distributions in the right column. For the spinning PLW the FTV+ responses clearly overweight the FTV- responses, indicating a strong FTV bias. For the PLW, FTV responses mainly occur at intervals 0º-90º, and 180º-270º. Since no correction of response times was applied the facing away responses (FTV-) have a narrow peak immediately after 90° and after 270°. This is likely due to a response delay resulting in mistakenly classifying a subset of the perceived FTV+ reversals as FTV- reversals. An FTV bias is also evident for the rigidly spinning human shape, but it is much weaker than for the PLW. No overall FTV or convexity-bias for the spinning half-cylinder is apparent.

**Fig 7:**
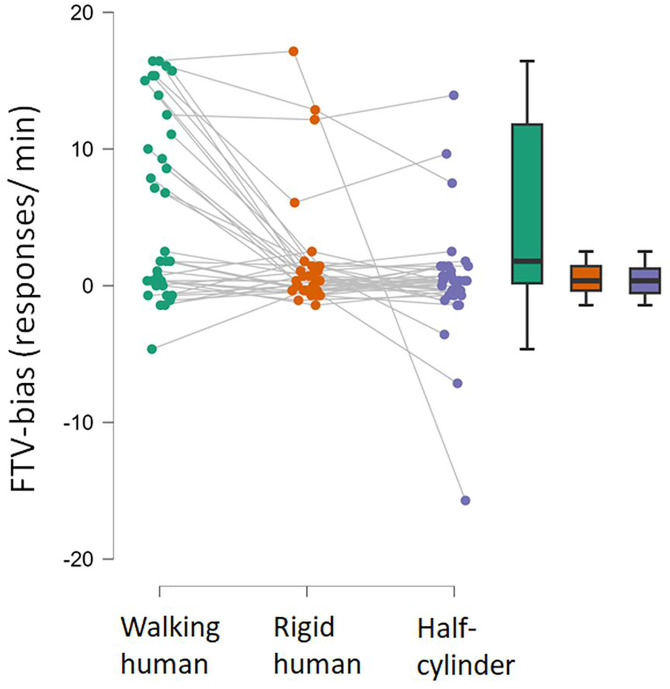
Facing the viewer bias. The FTV bias is scored as FTV- subtracted from the FTV+ responses for each participant, presented as a raincloud plot and the corresponding box-whisker plots showing the medium, the interquartile extent, and ranges excluding data beyond 1.5 times the interquartile ranges from the upper and lower bound of the boxes.

Fig 7 shows the FTV-responses (or convexity-responses) per minute on the left graph and the corresponding box-whisker plots of reversals/minute. These were calculated as the difference between FTV+ or bulging outward responses and FTV- or bulging inward responses for each of the three stimuli. The positive and negative FTV-responses respectively indicate facing toward (convexity) and facing away (concavity) responses. The wobbling cylinder does not have any defined facing or bulging direction, and was therefore excluded from the analyses of possible FTV biases. A one sample Wilcoxon signed-rank test with test value = 0 showed a clear positive FTV bias from the PLW (W = 492, p < .001, BF_10_ = 527). A complete FTV bias would lead to two reversals for every turn, corresponding to 17 FTV responses per minute, which match the observers experiencing fast reversals. Since the figures made 8.6 turns/minute this means that these participants actually perceived reversals just about every time the figure began to face away. An FTV bias, although much less pronounced, was also found with the rigidly spinning human shape (W = 390, p = .018, BF_10_ = 13), although there is a variability between individuals, three observers experienced rapid FTV+ reversals. No FTV bias, or convexity-bias, was obtained from the spinning half-cylinder (W = 334, p = .34, BF_10_ = .43). For the spinning half-cylinder a few participants experience a bulging inward bias (FTV-) whereas some others experience a bulging outward bias (FTV+). FTV-biases were statistically different between stimuli (repeated measures ANOVA: F(2)= 25, p < .001; BF_10_ = 1560). Post hoc comparisons show that the PLW (walking human) results in stronger FTV-bias than the other two stimuli (52 < posterior odds < 57), whereas no difference was obtained between the spinning rigid human and spinning half cylinder, (posterior odds = 0.23). An additional Friedman test was conducted due to non-normality of the data which confirmed a statistically significant difference in FTV-bias across conditions (χ²(2) = 10, *p* < .006). The non-parametric Conover’s post-hoc comparison test also confirmed that the PLW results in stronger FTV-bias than the other stimuli (both p’s = .023, corrected by Bonferroni-Holm method), whereas the difference between the spinning rigid human and half cylinder were non-significant (p = 1.0).

A binomial two-tailed test of the first response indicating a FTV+ or an FTV- percept, or a corresponding bulging outward or a bulging inward percept, revealed an initial bulging outward bias for the spinning half-cylinder (proportion FTV responses = .73, p = .014, BF_10_ = 6.6, using a test value = .5 for the proportions under H_0_). No such corresponding initial FTV-bias was obtained for the PLW or the rigid human shape (proportion initial FTV responses for the rigid human = .63, p = .2, BF_10_ = .63; proportion initial FTV responses for the PLW = .56, p = .42, BF_10_ = .30). The distribution of reversals for the spinning half-cylinder may indicate a newly found motion-transparency edge-in-front bias that overrides the convexity bias, as will be described in the discussion section.

The data file is available in the supplementary files as S1 File and S2 File.

## Discussion

Our findings show that perceptual reversal rates and directional biases vary systematically with object geometry and motion type. The spinning PLW produced by far the fastest reversals and exhibited a strong facing-the-viewer (FTV) bias, consistent with compelling orientation-specific reversals for the spinning human walking figure, as previously demonstrated [10]. In contrast, the rigidly spinning human figure yielded substantially fewer reversals and only a modest FTV bias, suggesting that the introduction of rigid 3D structure attenuates the orientation-specific reversals observed for the PLW. The spinning half-cylinder elicited a pronounced initial convexity bias, with observers preferentially perceiving the surface as bulging outward; once this initial interpretation was overcome, subsequent reversals aligned instead with an edge-in-front bias, indicating that this cue ultimately dominates the depth interpretation. Finally, the wobbling cylinder produced the fewest reversals of all stimuli and, critically, its reversal rates did not correlate with any of the other three stimuli, reinforcing previous reports that its dynamics engage perceptual mechanisms distinct from those driving reversals in spinning figures [9, 10].

The edge-in-front bias is consistent with previous reports showing a preference to perceive a kinetic surface edge as being in front of, rather than behind, another surface [35]. No evidence that depth symmetry facilitates reversals were obtained for the spinning human figure or the spinning half-cylinder. Although the perceived left and right spinning reversals were equal (due to the few unclear/mixed responses) a left duration bias was obtained for the spinning half cylinder and the wobbling cylinder, possibly due to a fixation preference where a fixated or attended part of a depth-ambiguous object is perceived as near [36]. While reversal rates for the spinning stimuli correlated with each other, those for the wobbling SFM did not correlate with the other SFM or PLW stimuli, generalizing the results from previous reports [8,10]. The correlation between spinning stimuli could be due to a common adaptation-recovery cycles It should be noted that for the rigidly spinning SFM stimuli, the perceived near and far elements exhibit opposing motion, in contrast, the spinning PLW deviates from this rule due to the pendular dynamics of its limbs. Still, observers readily perceive a walking human figure, suggesting that the visual system effectively decomposes the global rotational motion from the pendulum motions of the limbs. Finally, the FTV biases obtained with the spinning PLW and the rigidly spinning human figure correlated. Such correlations suggest that part of the processes producing reversals have a common origin.

Even though the ambiguous stimuli in this study were limited to motion-defined point-light asymmetric 3D structures, reversal rates differed substantially, as found in previous reports for motion defined ambiguous stimuli [8,10], and other types of ambiguous stimuli [17,37]. The spinning PLW resulted in faster reversal rates than the rigid SFM stimuli and much stronger FTV-bias than the rigidly spinning human shape. It was previously shown that the limb motion results in faster reversal rates for both an upright and an inverted spinning PLW, but only the upright PLW produce an FTV-bias [10]. These differences between an upright and an inverted spinning PLW, and corresponding rigidly spinning human shape, implies that while limb motion is essential for the increased reversal rate, the FTV bias is selectively activated by the upright, readily identifiable human form. The weak FTV bias observed here for the rigidly spinning human shape was driven by a few observers, and was absent in the previous study. It may be attributable to the use of a fixation cross above the stimuli, which was not employed previously. This parafoveal presentation, and accompanying lower visual resolution, could enhance the FTV bias for some observers compared to free viewing without a fixation cross. The PLW elicited a strong FTV bias, though the distribution of responses indicate bimodality: one group of observers rarely reported reversals, while another group experienced reversal rates of about twice for each 360° turn of the figure (see the raincloud plot in Fig 4), indicating a robust FTV bias.

The causes to the FTV bias have been debated, it may be a consequence of the convexity-bias [25,26,38] where attention is directed toward the knees which are preferentially perceived to bulge toward the viewer implying an FTV bias. The convexity bias is likely experience-based and arise as a consequence of prior higher occurrences of objects with outward bulging surfaces than with inward bulging surfaces. The fixation cross above the stimuli used here should have prevented any overt attention to the lower parts of the PLW. Still, a very strong FTV bias was observed that could result from covert attention to the lower part of the PLW. Another experience-based explanation for the FTV-bias is that in natural environments it is more critical to track, and focus on approaching than receding individuals [10]. Consequently, this could lead to an FTV bias. This explanation of the FTV bias is consistent with a cognitive strategy aimed at minimizing the risk of incorrectly perceiving an approaching person as receding to provide a link between observed correlation between social anxiety and the FTV bias, although the data to support this explanation seems inconsistent [39,40].

**Fig 8:**
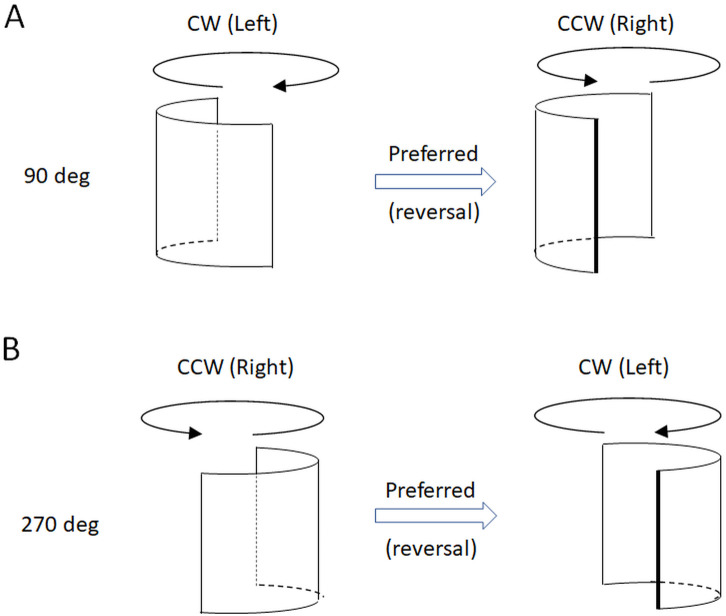
The two interpretations of the half-cylinder. **(A)** When bulging leftwards (90°) a counter clockwise (CCW) spinning is preferred. **(B)** When bulging rightwards a clockwise (CW) spinning is preferred (rightmost illustrations). During its phases in the spinning motion when an edge of the spinning half-cylinder begin to be self-occluded by its perceived near surface, it reverses in depth so that the edge is instead perceived moving in front. For illustrative purposes the half-cylinder is displayed with an oblique viewpoint slightly from above.

For the spinning half-cylinder stimulus, while an initial convexity bias was observed, neither the expected reversal pattern seen in hollow masks [29] emerged; instead, reversals clustered asymmetrically, with CCW responses favouring the 90° side view (bulging to the left) and CW response peaks occurring at 270° side view (bulging to the right), suggesting an overriding edge-in-front bias. A depth-symmetry bias, where reversals peak at side views of the spinning half-cylinder should occur with equal CCW and CW frequencies of reversals at these two side views. In contrast, the mean response vector of the CCW reversals pointed toward 90º, and whereas the CW response vector point toward 270º. The edge-in-front bias reflects a perceptual tendency to interpret occluding contours as foreground elements, particularly when edges begin to self-occlude, prompting depth reversals that favour the edge appearing in front of the surface (Fig 8). For other SFM stimuli, where kinetic edges are not so distinct, depth-asymmetric perspectives tend to resist abrupt reversals where previously empty visual regions become occupied, whereas at depth-symmetric perspectives reversals are smoother, and therefore more likely [16]. The preference of perceiving a kinetic edge in front [35] may be traced to the natural occurrences of edges mostly seen in front, and rarely through a front surface, and may be supported by lateral neural connections tuned to far depth planes [41], as well as attentional biases that render attended regions, such as contours, perceptually nearer.

The wobbling cylinder resulted in the lowest frequency of reversals among the stimuli used here and no asymmetry in the response distribution was obtained, which is in line with previous observations [8,10]. The wobbling cylinder is depth asymmetric throughout its motion which could account for the few and uniform distributions of reversals. Although both the wobbling SFM cylinder and a kinetic depth stimulus in the form of a similarly wobbling Necker cube are depth-asymmetric, their reversal rates do not correlate, and more frequent reversals are obtained with the wobbling Necker cube, suggesting different causes of the reversals for wire frame figures and point-light SFM [8]. The reversal rates for the wobbling cylinder did not correlate with reversal rates of any of the other stimuli used here, which generalizes the previous study.

Although bottom-up processes, such as adaptation and recovery cycles, have been shown to contribute to perceived reversals in spinning structure-from-motion (SFM) and Necker-type wireframe figures [13,30], top-down mechanisms are likely responsible for the various perceptual biases observed with the stimuli used here. Both shared and stimulus-specific endogenous processes appear to influence reversal rates across different forms of SFM stimuli. Notably, reversal rates increase significantly when observers are informed about the reversibility of the figures or instructed to switch perspectives rapidly, together with brain imaging, revealing that perceptual shifts are accompanied by cortical activity patterns consistent with top-down signalling, underscoring the role of top-down or cognitive influences [42–44]. Moreover, the top-down processes that drive perceptual reversals during observation of rigid SFM are selectively engaged when low-level influences, such as motion direction adaptation is minimized through the use of wobbling motion stimuli [8,10]. The spinning half-cylinder should also prevent efficient adaptation, as far and near direction-tuned units alternate in activation. Although this suggests that similar processes might operate for the wobbling cylinder and the spinning half-cylinder, it does not appear to be the case since no correlation between their reversal rates was observed.

The variability in the frequency of perceptual reversals between individuals likely result from differences in the balance of top-down versus bottom-up information processing, as shown in individuals diagnosed with ASD [18–20]. Interestingly, the variability in reversal rates is related to local variations in neurotransmitter activity [45], and may be linked to various personality traits [36]. Other biases may be due to the architecture of the neural hardware. An example is the bistable motion quartet whereby a 2D apparent motion stimulus in which four alternating dots arranged in a square can be perceived as moving either vertically or vertically. It reverses perceptually between these interpretations but with a vertical motion bias, which has been linked to the dominance of intra-hemispheric processing [12]. Among stimulus parameters not investigated here is spinning speed. Although Lissajous figures reverses preferentially in depth-symmetric perspectives, when spinning rapidly they do not reverse as frequently as when it is slowly spinning suggest that its momentum increases its stability [7].

Biological motion conveys much more information than non-living objects, and result in more wide-spread cortical activity compared to non-living objects [46–48]. Other evidence for different processes comes from detection threshold studies where the facing direction of a PLW when embedded in noise, is detected within a flexible time window, whereas the direction of a rigid human shape in a walking pose is integrated within a fixed time window [49]. This could be related to the discovery of a specific mechanism that detects the limb motions involved in biological motion, mediated by hypothesised life-detectors [50]. The ability to interpret biological motion is a crucial ability that may stem from intrinsic predispositions [51–53], or proprioceptive signals [54] resulting in embodied perception. The ability to recognize living creatures from point light displays is present in various species, including cats, dogs, fish, and even jumping spiders [55–57]. Further, biological motion perception has inspired explorations of the boundary conditions of biological motion perception [50,58–61], of brain areas involved [46–48], research on visual and social cognition [62,63], and personality and clinical psychology [64,65].

To conclude, we investigated how different ambiguous, motion-defined point-light stimuli elicit perceptual reversals and biases in human observers. The findings demonstrate that stimulus-specific shape and motion profiles jointly determine perceptual reversal dynamics. The biases influencing reversals are likely derived from past experiences of various shapes and motion types. Perceived reversal of the spinning human figures was influenced by the FTV-bias, and for the spinning half-cylinder reversals were influenced by the novel edge-in-front bias whereas the first impression revealed a convexity bias. No convexity bias, or depth-symmetry bias was obtained from the reported reversals, despite the fluctuations between convexity and concavity, and fluctuations in depth-symmetry of the spinning half-cylinder. The involvement of multiple processes and biases with various impact on different ambiguous stimuli influences theory development and attempts to identify the neural correlates of conscious experience [66]. These findings could refine models of multi-stable perception to involve multiple factors influencing perception of ambiguous stimuli, and informs research linking reversal rates of various ambiguous stimuli to individual traits and clinical conditions.

## Supporting information

S1 Movie Spinning walker. (MP4)

S2 Movie Spinning rigid human in walking pose. (MP4)

S3 Movie Spinning half-cylinder. (MP4)

S4 Movie Wobbling cylinder. (MP4)

S1 File Means. (CSV)

S2 File All responses. (CSV)
